# The anti-addiction drug ibogaine inhibits cardiac ion channels: a study to assess the drug’s proarrhythmic potential

**DOI:** 10.1186/2050-6511-13-S1-A38

**Published:** 2012-09-17

**Authors:** Xaver Koenig, Michael Kovar, Lena Rubi, Ágnes K Mike, Péter Lukács, Vaibhavkumar S Gawali, Hannes Todt, Walter Sandtner, Karlheinz Hilber

**Affiliations:** 1 Department of Neurophysiology and Neuropharmacology, Center for Physiology and Pharmacology, Medical University of Vienna, 1090 Vienna, Austria

## Background

The plant alkaloid ibogaine has shown promising anti-addictive properties in animals and humans. Although not licensed as a therapeutic drug, and despite evidence that ibogaine may disturb the rhythm of the heart, this alkaloid is used as an anti-addiction drug in alternative medicine. We have recently reported that therapeutic concentrations of ibogaine inhibit human ERG (hERG) potassium channels, and thereby uncovered a mechanism by which the drug may induce life-threatening cardiac arrhythmias.

## Methods

Here, to assess the drug’s proarrhythmic potential in more detail, we studied the effects of ibogaine and its congener 18-methoxycoronaridine (18-MC) on various cardiac voltage-gated ion channels by using the whole cell patch clamp technique. Besides heterologously expressed ion channels in TSA-201 cells, native channels in isolated mouse and guinea pig ventricular cardiomyocytes were also studied. Finally, we performed computer simulations to estimate drug effects on the human cardiac action potential (AP).

## Results

We confirmed that heterologously expressed hERG currents are reduced by ibogaine in low micromolar concentrations (IC_50_, 4 µM). Moreover, at higher concentration, the drug also reduced human Na_V_1.5 sodium currents. Experiments on mouse cardiomyocytes confirmed that ibogaine also inhibits voltage-gated ion channels in their native environment. 18-MC also reduced cardiac ion currents, but less potently than ibogaine. Although blocking hERG channels, ibogaine did not prolong the AP in guinea-pig cardiomyocytes at low micromolar concentrations. Higher concentrations (>10 µM) even shortened the AP. Finally, implementation of ibogaine’s inhibitory effects on ion channels in a computer model of a human ventricular cardiomyocyte suggested that calcium channel blockade by the drug counteracts the AP-prolonging effect generated by hERG inhibition.

## Conclusions

Because ibogaine inhibits cardiac ion channels in therapeutic concentrations, the drug is potentially proarrhythmic. The risk of its administration, however, is possibly reduced by the fact that the drug also shows antiarrhythmic properties.

